# Pain catastrophizing, kinesiophobia and fear-avoidance in non-specific work-related low-back pain as predictors of sickness absence

**DOI:** 10.1371/journal.pone.0242994

**Published:** 2020-12-10

**Authors:** Israel Macías-Toronjo, María Jesús Rojas-Ocaña, José Luis Sánchez-Ramos, E. Begoña García-Navarro

**Affiliations:** 1 Physical Therapy, Department of Rehabilitation, Huelva Fremap Hospital, Huelva, Andalucía, Spain; 2 Department of Nursing and Health Sciences, University of Huelva, Huelva, Spain; 3 Research Group ESEIS, Social Studies and Social Intervention, Center for Research in Contemporary Thought and Innovation for Development (COIDESO), University of Huelva, Huelva, Spain; Cairo University, EGYPT

## Abstract

The influence of pain catastrophizing, kinesiophobia and fear-avoidance attitudes towards non-specific low-back pain has been scarcely studied in an occupational insurance provider context. The objective of this work is to ascertain the relationship between these psychosocial variables with work absence, its duration and the disability of subjects with work-related low back pain. This is a descriptive observational methodological strategy. All patients with work-related non-specific low back pain who attended to an occupational health hospital during the study period were included consecutively. Clinical variables of kinesiophobia, pain catastrophizing, fear-avoidance attitudes, disability and pain were collected; sociodemographic variables of sex, age, type of work, educational level, occupational status and duration in days of work absence were recorded. Kinesiophobia (b = 1.43, *P* = 0.011, r = 0.333), fear-avoidance beliefs in its global dimension (b = 0.910, *P* = 0.014, r = 0.321), fear-avoidance beliefs in its work dimension (b = 1.255, *P* = 0.016, r = 0.321) and pain catastrophizing (b = 0.997, *P* = 0.013, r = 0.340) show individual association with the duration of sickness absence. Kinesiophobia (b = 0.821, *P* = 0.011, r = 0.30) and fear-avoidance beliefs (b = 1.760, *P* = 0.016, r = 0.28) are associated with disability (Kinesiophobia, b = 0.880, *P* = 0.045, r = 0.26; Fear-avoidance beliefs, b = 0.724, *P* = 0.010, r = 0.34). Kinesiophobia, fear-avoidance beliefs and pain catastrophizing are related to an increase in the duration of work absence and disability in patients with back pain in an occupational insurance provider context.

## Introduction

In recent years, the incidence of low back pain (LBP) has risen globally [1, 2]. The prevalence of an episode of LBP over a one-year period fluctuates between 22% and 65% [3], with the prevalence of acute LBP over an individual’s lifetime standing at 84%, and chronic LBP at 23% [4, 5]. In Spain, the prevalence of low back pain in 2006 was 19.9%, a figure that continues to increase progressively [6]. In an occupational context, the LBP is the main cause of disability due to work accidents [7], leading to increased health and social costs, as well as a considerable absenteeism due to occupational causes.

Globally, 37% of LBP prevalence could be attributed to factors related to the working environment [8]. Thus, conditions of an occupational nature such as mechanical work, bending and twisting postures, vibrations or lifting weights [9, 10]; job satisfaction, social coverage or high physical demand in the workplace are also associated with a higher incidence of LBP and would influence its prognosis [9, 11]. Therefore, interventions that impact on these factors have been shown to be effective in order to reduce the time to return to work, improve pain and disability in work contexts [12, 13].

At a psychosocial level, kinesiophobia and fear-avoidance beliefs are factors that play an important role in the evolution of the LBP and its transition towards chronicity [14, 15]. The Fear-Avoidance Model focuses on patients’ beliefs about disease, movement and pain by creating myths related to mistaken thoughts about the painful experience. According to this model, there would be an asynchrony between the disorder natural evolution and the clinical manifestation referred by the patient [16]. Avoidance and hypervigilance to pain behavior would be based on catastrophic thoughts that activate limiting attitudes, which turn, would amplify disability and pain [15, 16]. Thus, catastrophic thoughts are related to the fear of movement and this, in turn, to worse outcomes on therapeutic interventions [17–19].

Oliveira *et al*. insist on the identification of psychosocial risk factors within a multidisciplinary approach in the management of patients with LBP [20]. Fear of movement and pain catastrophizing as an influence on sickness absence and return to work have been scarcely studied in an occupational insurance provider context. There are published studies that analyze low back pain from the point of view of primary care [21–24]. However, the literature is lacking in reports that take the perspective of an occupational insurance provider, taking into account that, in many countries, occupational accidents are specifically managed by these institutions. These are private non-profit organizations that collaborate with the public social security system to manage the economic and health issues of work accidents and occupational diseases. In these institutions, health monitoring and control are considered specific and agile in terms of resources such as specialist medicine, imaging tests or physical therapy treatments in comparison with public services that make this manuscript unusual in the analysis of non-specific LBP psychosocial factors.

Considering that the presence of psychosocial variables condition the evolution of low back pain disorders, these variables are related to the sickness absence status and its duration in work-related low back pain disorders, as well as the degree of disability reported by patients. Therefore, the purpose of this study is to determine the association of kinesiophobia, fear-avoidance beliefs and pain catastrophizing as psychosocial conditions with sickness absence, its duration and disability in work-related low back pain. This research would be justified by the need to identify these non-strictly clinical variables of such prevalent disorder as low back pain in an occupational setting, to be identified and taken into account in guidelines and multidisciplinary therapeutic approaches to occupational accidents.

## Materials and methods

### Type of study

The methodological strategy was planned with a descriptive observational design, placing the study in the Clinical Health Service of an occupational insurance provider.

### Subjects

The subjects of the study were individuals with LBP who presented to an occupational health clinic with a diagnosis of non-specific low back pain due to a work accident between 1 June 2018 and 31 December 2019. Non-specific pain is considered to be pain that is not caused by fractures, direct trauma, or systemic disease and where there is no proven root compression amenable to surgical treatment [24]. A work-related accident is any bodily injury that the employed worker suffers on the occasion or as a result of work. The target population was the entire group of patient who met the inclusion criteria. All patients (n = 88) who met this inclusion criteria were consecutively included.

The following inclusion and exclusion criteria were applied:

Inclusion criteria:

1. Work-related nonspecific LBP attending to an occupational health clinic.
2. Age between 18–65 years old.
3. Understanding the language, informed and signed consent.

Exclusion criteria:

1. Back pain related to infection, cancer, fracture, visceral disease, spondylarthrosis, extruded disc herniation or cauda equina syndrome.
2. Previous treatment for spinal pain.
3. Previous surgical intervention, commuting accident, common illness or occupational disease.
4. Cognitive impairment.

The same day of the work accident, injured patients were attended by the occupational insurance medical service and, after diagnosis and typification as a work accident, it was noted whether patients were on sickness absence status or whether they could reconcile their back pain process with his professional activity. At the end of their visit to the physician, each patient who met the eligibility criteria was informed of the study objectives and was asked to participate. All patients included in the study signed the relevant informed consent form. The participants knew that the information collected was confidential and anonymous.

This first day of attendance, the participants were also interviewed by the physiotherapist in charge where sociodemographic information was collected. The content of the questionnaires was explained and completed only once, prior to any other therapeutic intervention, that same day. The tracking of the duration of the sickness absence was measured in the days off from the day of the work accident until the patients returned to their job. Any re-injury or setback of the same LBP disorder after the six months after the day of return to work were considered as part of the same process of sick leave and were thus recorded as the same sick absence. Any change in the initial diagnosis of nonspecific low back pain of any of the participants throughout the follow-up process would cause them to not meet the inclusion criteria and be excluded from the study.

This study followed the ethical principles for medical research in human beings according to the Declaration of Helsinki and the protection of data and guarantees of digital rights according to organic law 3/2018 of December 5, 2018. The study was authorized by the Fremap Mutual Ethics Committee (Code number FREMAP-2200631-Z).

### Study variables

#### Clinical variables

*Tampa Kinesiophobia Scale (TSK)*. Fear of movement is considered a predictor in terms of perpetuation of pain and one of the most important constructs of the Cognitive-Behavioral Model of fear avoidance. The Tampa Scale of Kinesiophobia is one of the main instruments on which this model is based to measure fear of movement [25]. In this study, the TSK-11 items was used, which demonstrated similar validity and reliability as the original TSK-17 items [26, 27]. The Spanish version of the scale has been validated for patients with both chronic and acute pain [25] (Table 1). The scale consists of 11 items and each item is scored based on a 4-point Likert scale, ranging from “strongly agree” (4 points) to “strongly disagree” (1 point). The score ranges from 11 to a maximum of 44, with the highest scores indicating strong fear of movement or fear of re-injury from movement.

**Table 1:** Instrument properties used for non-specific LBP.

Intruments	Intraclass Correlation	Reliability. Internal Consistency	Validity
**Roland Morris Questionarie**	ICC^a^ = 0.874 (95% confidence interval)	Cronbach's α = 0.8375 (day 1) and 0.9140 (day 15).	Concurrent validity r = 0.347 (*P* <0 .01) for day 1, and r = 0.570 (*P* < 0.01) for day 15.
			Construct validity r = 0.197 (*P* = 0.0061) on day 1 and r = 0.341 (*P* < 0.01) on day 15^b^
**Fear Avoidace beliefs**	ICC^a^ = 0.967 (95% confidence interval)	Cronbach's α = 0.9337	R_s_ = 0.522 (*P* <0 .0001); R_s_ = 0.320 (*P* <0 .0001) on day 1; R_s_ = 0.637 (*P* <0 .0001); R_s_ = 0.457 (*P* <0 .0001) on day 15^c^
**Tampa Scale Kinesiophobia**	ICC^a^ = 0.85	Cronbach’s α = 0.79 for chronic pain; Cronbach’s α = 0.81 for acute pain	Construct validity, r = 0.34 (*P* < 0.01); r = 0.34 (*P* <0 .01)
			Predictive Validity, r = 0.46 (*P* < 0.001); r = 0.46 (*P* < 0.001); r = 0.29 (*P* < 0.01)^d^
**Pain catastrophizing Scale**	ICC^a^ = 0.84 (95% confidence interval)	Cronbach α = 0.82 for athletes and Cronbach α = 0.79 for patients with fibromyalgia	Construct validity, r = 0.44 (*P* < 0.01); r = 0.68 (*P* < 0.01)^e^

^a^Intraclass Correlation Coefficient.

^b^Tested by determining the correlation between the Spanish version of Roland Morris Questionnaire and the Spanish version of the Oswestry Questionnaire.

^c^Tested by determining the correlation between Fear avoidance Beliefs and Spanish Roland Morris Questionnaire and Referred Pain.

^d^Tested by determining the correlation between Tampa Scale Kinesiophobia and Pain Catastrophizing Scale and Hospital Anxiety and Depression Scale.

^e^Tested by determining the correlation between the Spanish version of Pain catastrophizing Scale and Hospital Anxiety and Depression Scale and Fear Avoidance beliefs.

*Pain Catastrophizing Scale (PCS)*. Pain Catastrophizing is considered an important prognostic factor in chronic LBP but it is also used in patients with acute pain [22]. The pain catastrophizing scale is a 13-item instrument that measures on a 5-point scale the extent to which subjects develop certain feelings and thoughts related to their nociceptive experience [28]. This instrument, which has a validated version for Spanish [29, 30] shows an adequate internal consistency (Table 1).

*Fear-Avoidance Questionnaire (FABQ)*. Fear-avoidance beliefs about work are closely related to work LBP disability [31]. The questionnaire reflects how physical and occupational activity influence the nociceptive experience [31]. It consists of two parts, one on fear-avoidance beliefs during work activity (Cronbach’s alpha = 0.88) and another on physical activity (Cronbach’s alpha = 0.77). The Spanish version of the fear-avoidance beliefs questionnaire has good comprehensibility, consistency and reliability, with the full version being as valid as both subscales, as well as being easier to score and analyze thus facilitating its use in clinical practice [32]. In this study, the full scale was used (Table 1).

*Roland Morris Disability Questionnaire (RMQ)*. Disability was measured using the Roland Morris Questionnaire instrument. It is one of the world’s best validated and globally used scales to measure LBP disability. It consists of 24 items related specifically to daily physical activities likely to be affected by LBP scoring between 0 (no disability) and 24 (maximum disability). A Spanish version has been validated and has become a reliable instrument to evaluate disability in patients with LBP [33], with an intraclass correlation of 0.87, good concurrent and construct validity and a high internal consistency (Table 1). Disability is expressed in absolute values.

*Numerical Pain Scale*. The Numerical Pain Scale was used to measure pain intensity [34]. On an 11-item scale, 0 indicates an absence of pain while 10 represents the worst imaginable pain.

#### Socio-demographic variables

Sociodemographic variables were collected on sex, age, occupational and educational level, as well as data on sickness absence status and duration of sickness absence in days.

The 2011 Spanish National Occupations Classification put together by the Spanish Office for National Statistics, was used for the “type of work” variable. This classification rates occupations according to the level of competences required. It includes 4 levels [35]:

- Competence level 1. Occupations under competence level 1 usually entail the performance of simple and routine physical or manual tasks. Use of manual tools may be required.
- Competence level 2. Occupations under competence level 2 usually require performing tasks such as machine and electronic equipment operation, driving of vehicles, maintenance and repair of electrical and mechanical equipment, and handling, organizing, and sorting information.
- Competence level 3. Occupations under competence level 3 tend to encompass technical tasks and complex routines that require different kinds of technical and practical knowledge specific to a certain subject.
- Competence level 4. Occupations under competence level 4 require the performance of tasks involving decision-making and complex problem-solving based on a profound theoretical and practical understanding of a certain subject matter.

For the educational level variable, subjects were assigned to different groups depending on whether they had completed primary education, secondary education, pre-university education, or university education.

### Statistical analysis

Initially, a descriptive analysis was made of the variables studied. In order to analyze the relationship between variables, after a description, a bivariate analysis of the variables associated with sickness absence status, its duration and the degree of disability reported was carried out. The relationship of the socio-demographic variables (sex, education and occupation levels) with sickness absence status was analyzed using the chi-square statistic, once the conditions of application had been verified. The level of professional competence was recoded, grouping the only level 4 case with level 3 cases. After checking graphically the absence of deviations from normality by means of Q-Q charts without trends and the homogeneity of variances, the means of the variables by means of the student´s T test to see the differences between patients who had presented sickness absence status and those who had not was compared. The association of clinical variables with the duration of sickness absence and the degree of disability was evaluated using a linear regression model, controlling for the variables occupation and educational level. The association of kinesiophobia, pain catastrophizing, fear-avoidance beliefs and pain intensity level variables with the duration of sickness absence and the level of disability reported was verified using multiple linear regression, controlling for socio-demographic variables that could act as confounding factors.

## Results

The present study includes 88 subjects with work-related low back pain for whom 58 were men (65.9%) and 30 women (34.1%). Subjects with primary education (58%) were in the majority. Other educational levels accounted for decreasing proportions of the sample, with subjects with a university education constituting the smallest group (6.8%). A similarly decreasing pattern was observed in the realm of occupational competence, where 75 subjects (82%) were classified as level 1 and 7 subjects as level 3 (8.0%). Work-related LBP usually resulted in sickness absence, with 68.2% of the subjects being in a sickness absence status for at least one day compared to 31.8% who reported to work in spite of their condition. The follow-up was made independently on each patient sickness absence until the return to work and all subjects returned to their job. There was, therefore, no drop out for this reason in any of the participants in the follow-up. Mean age of the participants was 41.6 years (SD = 8.40). Mean duration of sickness absence was 17.71 (23.02) days. In most cases, the degree of disability declared at the first medical contact was 46.64%. The mean score on the Tampa Kinesiophobia scale was 30.20 (7.43). The mean score on the pain catastrophizing scale was 27.35 (11.82) for all subjects. The mean score on the fear-avoidance scale was 44.67 (12.71) in the overall dimension, 17.85 (5.79) in the physical activity dimension and 26.81 (9.03) in the work dimension. The mean score on the numerical pain scale was 7.09 (1.88) for all subjects.

### Sickness absence status

The educational and occupational level sociodemographic variables appeared to be related to LBP sickness absence variable (Table 2). Compared to having a university education, those with a secondary education had 2.75 times higher chance of going on sickness absence (IC95%: 0.40–18.88), and those with a primary level of education had 8.20 times higher chance of going on sickness absence (IC95%: 1.31–51.25). Subjects with a primary competence level are 3.67 times more likely to be in a sick absence status than those with occupational level 3 (IC95%: 0.75–17.84). Sex was not associated with being on sickness absence according to the data (Table 2). Nor did the age variable seem to be associated with sickness absence; subjects on sickness absence had a mean age of 42.35 years (8.21) and a mean age of 40.07 years (8.86) those who were not on sickness absence. Occupation and education variables are considered confounding variables and were used as control for clinical variables. Clinical variables do not seem to be directly related to the sickness absence status (Table 3).

**Table 2:** Frequency of sickness absence in each sociodemographic group^a^.

Variables	Sickness Absence (n)	Sickness Absence (%)	*P*†	Comparison	Odds Ratio	Inferior Limit CI95%	Superior Limit CI95%
**Sex**							
Males	37	63.8	0.219	Men/women	0.54	0.20	1.46
Females	23	76.7					
**Education**							
Primary	41	80.4	0.022	Primary/University	8.20	1.31	51.25
Secondary	11	57.9		Secondary/University	2.75	0.40	18.88
PreUniversity	6	50.0		PreUniversity/University	2.00	0.26	15.38
University	2	33.3					
**Occupation**^**b**^							
Level 1	55	73.3	0.042	Level 1/Level 3–4	3.67	0.75	17.84
Level 2	2	33.0		Level 2/Level 3–4	0.67	0.07	6.41
Level 3–4	3	42.9					

^a^Performing a sensitivity analysis in which the sickness absence status variable is required to last a minimum of 4 days, the association between sociodemographic and sickness absence status variable is maintained, as presented in S1 Table.

^**b**^Occupations: Level 1: Simple and routine physical. Level 2: Handling of machinery, electronic equipment, storing and sorting information. Level 3: High educational level, advanced communication skills, ability to understand complex written materials. Level 4: performance of tasks involving decision-making and complex problem-solving based on a profound theoretical and practical understanding of a certain subject matter.

† Chi-square test.

**Table 3:** Mean values for the different clinical variables as a function of the presence (yes) or absence (no) of sickness absence status.

Sickness Absence	Yes			No			*P*‡
	n	Mean	SD	n	Mean	SD	
**Catastrophizing Scale**	60	28.45	11.28	28	25.00	12.80	0.204
**Kinesiophobia Scale**	60	31.21	7.71	28	28.28	6.49	0.085
**Fear Avoidance Scale**	60	46.40	12.27	28	40.96	13.07	0.061
**Fear Avoidance Work**	60	27.80	8.46	28	24.71	9.98	0.136
**Fear Avoidance Physcial Activity**	60	18.60	5.58	28	16.25	5.99	0.076
**Pain Intensity**	60	7.27	1.54	28	6.71	2.42	0.198

‡ t-Student.

### Duration of sickness absence

As regards the duration of the sickness absence and its relationship with socio-demographic variables, none of the socio-demographic variables studied (occupation (*P* = 0.435), education (*P* = 0.222) and sex (*P* = 0.859)) are related with the duration of sickness absence variable. Kinesiophobia (b = 1.43, *P* = 0.011, r = 0.333), fear-avoidance beliefs in its global dimension (b = 0.910, *P* = 0.014, r = 0.321), fear-avoidance in its work dimension (b = 1.25, *P* = 0.016, r = 0.321) and pain catastrophizing (b = 1.00, *P* = 0.013, r = 0.340) show individual correlation with duration of sickness absence once controlled confounding variables in linear regression (Table 4).

**Table 4:** Linear multiple regression of sickness absence duration variable with individual clinical variables controlled by education and occupational confounding factors.

Duration of sickness absence	b	95% C.I. (b)		r	*P*
		Lower	Upper		
**Catastrophizing Scale**	1.00	0.22	1.77	0.34	0.013
**Kinesiophobia Scale**	1.43	0.34	2.52	0.33	0.011
**Fear Avoidance Scale**	0.91	0.18	1.55	0.32	0.014
**Fear Avoidance Work**	1.25	0.24	2.26	0.32	0.016
**Fear Avoidance Physical Activity**	1.35	-0.18	2.87	0.23	0.082
**Pain Intensity**	2.08	-3.62	7.78	0.01	0.468

### Disability

As regards disability, education and occupation variables appear again related (Table 5). The different clinical variables show an individual association with low back disability in an initial model. In a joint statistical model, kinesiophobia (b = 0.82, *P* = 0.011, r = 0.30) and fear-avoidance beliefs (b = 1.76, *P* = 0.016, r = 0.28) are associated in multiple linear regression, both in the overall of the subjects and in subjects on sickness absence, with the disability reported in the first medical contact, with the control of the confounding variables (Kinesiophobia, b = 0.88, *P* = 0.045, r = 0.26; Fear-Avoidance, b = 0.72, *P* = 0.010, r = 0.34) (Table 6).

**Table 5:** Relationship between low back disability and sociodemographic variables. Comparison of means.

Disability		n	MEAN	SD	*P*†
**Occupation**	Level 1	75	49.33	25.62	0.042
	Level 2	6	27.08	21.85	
	Level 3	7	34.52	27.40	
**Education**	Primary Education	51	50.16	24.93	0.035
	Secondary Education	19	48.03	26.07	
	Pre-University Education	12	37.85	28.84	
	University Education	6	29.86	26.80	
**Sex**	Man	58	43.17	26.32	0.084
	Woman	30	53.33	24.77	

† Chi-square test.

**Table 6:** Multiple linear regression between the declared LBP disability and the scales of kinesiophobia, fear avoidance behavior, pain intensity and catastrophizing.

a) Initial models, adjusted for education and occupation level					
	**b**	**95% C.I. (b)**		**r**^**a**^	***P***
		**Lower**	**Upper**		
**Catastrophizing Scale**	0.82	0.34	1.31	0.37	0.001
**Kinesiophobia Scale**	1.76	1.10	2.42	0.50	<0.00001
**Fear Avoidance Beliefs**	1.03	0.62	1.43	0.50	<0.00001
**Fear Avoidance Work**	1.13	0.53	1.72	0.39	<0.00001
**Fear Avoidance Physical Activity**	2.05	1.18	2.92	0.45	<0.00001
**Pain Intensity**	3.55	0.58	6.52	0.25	0.020
b) Final model adjusted for educational and occupational level.					
	**b**	**95% C.I. (b)**		**r**^**a**^	***P***
		**Lower**	**Upper**		
**Catastrophizing Scale**	1.13	-0.32	0.68	0.81	0.478
**Kinesiophobia Scale**	0.82	0.25	1.84	0.30	0.011
**Fear Avoidance Beliefs**	1.76	0.11	1.04	0.28	0.016
**Pain Intensity**	1.03	-1.06	4.23	0.11	0.237
c) Final model adjusted for Education and Occupation and for the remaining variables in the model for patients on sickness absence.					
	**b**	**95% C.I. (b)**		**r**^**a**^	***P***
		**Lower**	**Upper**		
**Catastrophizing Scale**	0.27	-0.33	0.88	0.12	0.363
**Kinesiophobia Scale**	0.88	0.02	1.74	0.26	0.045
**Fear Avoidance Beliefs**	0.72	0.18	1.27	0.34	0.010
**Pain Intensity**	3.10	-0.46	6.67	0.19	0.086

a) individual regressions for each variable; b) final model: multiple regression that includes all clinical variables; c) multiple regression that includes all clinical variables in patients on sickness absence. All models are controlled by education and occupation levels.

^**a**^Partial correlation.

## Discussion

The objective of this study is to determine the relationship between the psychosocial variables of kinesiophobia, fear-avoidance beliefs and pain catastrophizing on sickness absence status and its duration, as well as on the disability declared in first attendance in a context of work-related non-specific LBP in a specific occupational insurance provider. These clinical variables have been controlled by sociodemographic variables of educational and occupational level considered as possible confounding variables.

### Pain catastrophizing

The results establish an intense relationship between psychosocial variables of pain catastrophizing, fear avoidance beliefs and kinesiophobia with the duration of the sickness absence and the disability declared, but not with the sickness absence status. The typification of the sickness absence status would not be related to personal pain coping strategies, but rather to an assessment by health personnel about the subject's ability to carry out their work activity.

Ramírez-Maestre et al. have already pointed to pain catastrophizing as the central axis in the relationship of psychosocial variables with disability, following a model in which kinesiophobia and fear-avoidance behaviors intervene as mediators in this relationship [22]. The degree of intensity of the results allows us to establish a strong association of pain catastrophizing as part of the recovery process and its influence on the prognosis of LBP. As well, Wertli et al., in a meta-analysis, also link pain catastrophizing and coping strategies with disability and pain intensity, but the relationship with the duration of sickness absence and return to work would not be clear [36]. In this case, these results contrast partially with this research, in which all psychosocial variables studied are strongly associated with disability and with the duration of sickness absence. In a context of a specific occupational insurance provider, where return to work time and the occupational characteristics are increasingly evaluated, pain catastrophizing is a variable to be taken into account when identifying patients at risk of chronification and long-term absenteeism. In an occupational setting, economic compensations associated with sickness absence managed by the insurance provider or the sickness absence status itself could be related to these coping strategies and that, in turn, could influence the results of therapeutic approaches and recovery times [37–39]. However, most of the subjects in the sample belonged to the primary sector where temporary employment predominates and a long-term leave could condition work stability [40].

Also in line with this analysis, but in a context of primary care in the Spanish public health system, pain catastrophizing would be placed as a differential predictor of the evolution of LBP processes and associated with disability in a broad observational study on subacute and chronic low back pain [41]. Likewise, Løchting et al. relate pain catastrophizing and disability with the results of the treatments carried out on subjects with LBP [42]. Again, the strong association found between the variables studied could have an influence on the recovery processes of patients with non-specific lumbar pain and mediate therapeutic interventions, whether in pharmacologic treatment, active rehabilitation or orientation of lifestyle or postural habits in an occupational insurance provider context.

On the other hand, Kovacs et al., also in a three-month prospective study, do not associate pain catastrophizing with the recovery times of LBP disorders, but do point to the intensity of the pain as a predictive value of its evolution, being this study on subjects from the Spanish National Health System [43]. This issue contrasts sharply with the results, where even pain declared in first attendance would not be related to the duration of the sickness absence. Compared to public health services, the occupational insurance medical service offers a higher health response capacity, since the therapeutic approach is more specific on the work-related accident. Resources such as specialist medical appointments, complementary imaging tests, physical or rehabilitative treatment or, if invasive treatment is necessary (infiltrations or surgical treatment) is carried out early compared to public services.

### Fear-avoidance behaviors and kinesiophobia

Kinesiophobia and fear-avoidance attitudes are associated, both in the global of the subjects and in a context of sickness absence, with the disability reported and with the duration of the sickness absence. This pain coping strategies have been related in previous studies to disability and time to return to work [21, 23] but not to the sickness absence status, something in line with the results presented. In this sense, Diaz-Cerrillo et al., in a cross-sectional study and in a context of chronic pain, associate the sickness absence status with any of the scales of fear-avoidance beliefs, being the sickness absence status the factor that would independently explain increases in these scales, but not being associated with the declared disability [44], establishing a relationship between the sickness absence status and fear-avoidance beliefs that this work does not refute. The results would indicate that fear-avoidance and kinesiophobia behaviors would not be related to the sickness absence status typification of the patient injured, but could influence the duration of the leave, where the patient himself would manage the pathological process through personal coping pain strategies.

Similary, Henemeer et al. showed no relationship between kinesiophobia and fear-avoidance behaviors and sickness absence [45]. According to these authors, only pain intensity was related to disability and sickness absence status in subjects with chronic LBP. These author´s results partially contrast with this work, where clinical variables of kinesiophobia and fear-avoidance beliefs were related to both duration and disability, and pain intensity did not play a relevant role in determining the sickness absence status, its duration or the declared disability. In this sense, the fact that the data was collected on the same day of the accident could justify the high levels of pain reported and, in turn, could condition its relationship with the variables studied.

In the same line, Gheldof et al., indicate that kinesiophobia and fear of re injury are factors that explain disability in LBP beyond the intensity of back pain. Thus, the influence of kinesiophobia on disability would be more important than workload, work stress or work satisfaction, being fear of pain attitude a mediating factor between negative affectivity and disability [46]. For its part, other authors, also in a context of acute pain and using the Tampa scale of kinesiophobia, chronologically places the fear of movement in the first moments of an episode of low back pain and strongly related to the functional status, disability and ability to perform a task [47], identifying the fear of movement after a LBP process as a mediator of functionality and disability. Again, this work cannot place chronologically any of the variables studied, but it can confirm their importance within the work-related low back pain disorder in its relationship with absenteeism and disability.

Among the strengths of this study, it should be noted that the relationship between pain coping psychosocial variables and sickness absence status, disability and time to return to work described by this work has been scarcely studied in an occupational health insurance provider context. In addition, the selection of patients as well as the measurement and collection of the data was carried out the same day of the work accident with a follow-up of the subjects from the day of the work accident until the day of return to work. In this sense, the literature is lacking in reports where the period of time between the work accident, selection and data collection was so early and the results obtained could have been influenced by this early measurement, prior to any therapeutic intervention, and should be taken into account when developing low back pain treatment strategies that include these psychosocial variables.

### Study strength and limitations

Regarding the limitations of this work, although this cross-sectional study followed the duration of sickness leave among the patients in the sample, it does not warrant the establishment of a cause-effect relationship with respect to the variables analyzed. In this sense, longitudinal studies would be recommended. The authors of the present study hope that their findings may encourage further studies on the same topic with larger cohorts, and could include subjects from different work backgrounds.

## Conclusion

In an occupational accident context, where the occupational insurance provider, in collaboration with the different health systems, controls the monitoring of these LBP processes and where this monitoring is usually more specific and agile, these not strictly clinical variables seem to be related to recovery and duration of sickness absence and should be taken into account in the therapeutic approaches with the aim of reducing the time of occupational disability. Psychosocial factors of pain catastrophizing, kinesiophobia and fear-avoidance behaviors are related to the duration of sickness absence and the disability reported in non-specific LBP disorders in a context of occupational insurance provider. Kinesiophobia and fear-avoidance beliefs are strongly related to disability in first assistance. The psychosocial variables studied are not related to sickness absence status.

Considering that LBP is one of the most prevalent and disabling pathologies at work environment and its social impact, these variables, that are rarely identified in a context of recovery from pathology derived from an occupational accident and that would be strongly associated with disability and recovery times, should be included in treatment guidelines to facilitate their knowledge by health professionals and to speed up recovery times and return to work, prevent long-term disability in order to avoid greater absenteeism due to non-specific LBP.

## Supporting information

S1 Table Relationship between sickness absence greater or equal to 4 days and sociodemographic variables. ^**a**^Occupation: Level 1: Simple and routine physical. Level 2: Handling of machinery, electronic equipment, storing and sorting information. Level 3: High educational level, advanced communication skills, ability to understand complex written materials. Level 4: performance of tasks involving decision-making and complex problem-solving based on a profound theoretical and practical understanding of a certain subject matter. † Chi-square test.
(DOCX)
