# Supplementary material for: Pain catastrophizing, kinesiophobia and fear-avoidance in non-specific work-related low-back pain as predictors of sickness absence
Source: PLoS One. 2020 Dec 10;15(12):e0242994. doi: 10.1371/journal.pone.0242994 (PMC7728279; doi:10.1371/journal.pone.0242994)
Supplement: S1 Table — aOccupation: Level 1: Simple and routine physical. Level 2: Handling of machinery, electronic equipment, storing and sorting information. Level 3: High educational level, advanced communication skills, ability to understand complex written materials. Level 4: performance of tasks involving decision-making and complex problem-solving based on a profound theoretical and practical understanding of a certain subject matter. † Chi-square test. (DOCX) [file pone.0242994.s001.docx]

**S1 Table. Relationship between sickness absence greater or equal to 4 days and sociodemographic variables.**

| **Variables** | | **Sickness Absence ≥ 4 (n)** | **%** | ***P*†** |
| --- | --- | --- | --- | --- |
| **Sex** | Males | 35 | 60.3 | 0.227 |
|  | Females | 22 | 73.3 |  |
| **Education** | Primary | 40 | 78.4 | 0.006 |
|  | Secondary | 10 | 52.6 |  |
|  | Pre-university | 6 | 50.0 |  |
|  | University | 1 | 16.7 |  |
| **Occupation^a^** | Level 1. | 53 | 70.7 | 0.021 |
|  | Level 2. | 2 | 33.3 |  |
|  | Level 3-4. | 2 | 28.6 |  |

**^a^**Occupation: Level 1: Simple and routine physical. Level 2: Handling of machinery, electronic equipment, storing and sorting information. Level 3: High educational level, advanced communication skills, ability to understand complex written materials. Level 4: performance of tasks involving decision-making and complex problem-solving based on a profound theoretical and practical understanding of a certain subject matter. † Chi-square test.
